# ERICA: Study of Cardiovascular Risk Factors in Adolescents

**DOI:** 10.1590/S01518-8787.201605000SUPL1ED

**Published:** 2016-02-02

**Authors:** Rosely Sichieri, Marly A Cardoso

**Affiliations:** I Departamento de Epidemiologia. Instituto de Medicina Social. Universidade do Estado do Rio de Janeiro. Rio de Janeiro, RJ, Brasil; II Departamento de Nutrição. Faculdade de Saúde Pública. Universidade de São Paulo. São Paulo, SP, Brasil

Surveys conducted in several countries indicate increased level of blood pressure in adolescents, as well as the prevalence of hypertension in this group, with estimates around 10.0%.^2^ This growing prevalence of hypertension in young people stems mainly from the increase of obesity, observed in most countries^3^. However, the disease is also, independently, associated with the growing consumption of salt and sugar and other modern constraints such as stressful environments, low physical activity, and sedentary lifestyle^2^. In Brazil, a cross-sectional study on food consumption of Household Budget Survey 2008-2009 in representative sample of the population aged 10 or more years noted that the largest consumption of ultra-processed food was associated with higher content of fats in general, saturated fat, trans fat and free sugar. Less consumption of fiber, protein, sodium, and potassium was observed when compared to the fraction of consumption regarding *in natura* or minimally processed foods^5^.

This supplement of the *Revista de Saúde Pública* presents data from the Study of Cardiovascular Risks in Adolescents (ERICA), which allow us to assess the prevalence of hypertension and cardiovascular risk factors in Brazilian adolescents.

The data are of undeniable importance for the planning and evaluation of public policies and also for the development of more specific actions related to the school environment and to primary health care, aiming at the reduction of several injuries described to adolescents, ranging from inadequate food consumption to smoking and alcohol experimentation.

The importance of interventions in this phase of the life cycle is due to the large physiological changes that adolescents undergo, as occurs in body composition, with body fat gain among girls and muscle mass twice more expressive among boys^7^. These characteristics demand nutritional adequacy and psychological support for proper growth and development. In addition to these physiological changes, adolescents have great chance to acquire inappropriate eating habits and, in different countries, unhealthy dietary practices were observed in this age group. Therefore, adolescents feature high consumption of processed foods, including snacks, cookies, sugary drinks and other foods with high caloric density, besides low consumption of fruits and vegetables, healthy eating markers^4^ ^,^ ^8^.

Additionally, adolescents have been changing progressively the consumption of meals, frequently omitting breakfast and substituting lunch and dinner for unhealthy snacks, behavioral changes considered unfavorable to health^1^ ^,^ ^6^.

The articles in this Supplement assess the prevalence of hypertension and overweight in adolescents, as well as other cardiovascular risk factors, highlighting food consumption and physical activity features. Results were stratified according to regions of the country, school type, and cities’ characteristics.

This Supplement presents essential readings for scholars of chronic diseases and adolescence. It also allows a glimpse, by the amount and quality of the data obtained, of a material about adolescents’ health for more detailed future studies, which will enable the necessary and urgent measures for the prevention of chronic non-communicable diseases and injuries that begin in adolescence and persist into adulthood. Additionally, ERICA gathered a large numbers of researchers who will probably work on more appropriate living conditions for adolescents.
