# Supplementary material for: ERICA: Study of Cardiovascular Risk Factors in Adolescents
Source: Rev Saude Publica. 2016 Feb 2;50(Suppl 1):1s. doi: 10.1590/S01518-8787.201605000SUPL1ED (PMC4767029; doi:10.1590/S01518-8787.201605000SUPL1ED)
Supplement: Translation

# Translation: ERICA: Estudo dos Riscos Cardiovasculares em Adolescentes

**Authors:** Rosely Sichieri, Marly A Cardoso

**Affiliations:** I Departamento de Epidemiologia. Instituto de Medicina Social. Universidade do Estado do Rio de Janeiro. Rio de Janeiro, RJ, Brasil; II Departamento de Nutrição. Faculdade de Saúde Pública. Universidade de São Paulo. São Paulo, SP, Brasil

Pesquisas realizadas em vários países indicam aumento do nível de pressão arterial em adolescentes, assim como da prevalência de hipertensão nesse grupo, com estimativas ao redor de 10,0%^2^. Essa crescente prevalência global da hipertensão em jovens decorre principalmente do aumento da obesidade, observada na maioria dos países^3^. Entretanto, também associa-se de forma independente com o crescente consumo de sal e de açúcar e com outros condicionantes do estilo de vida moderno, como ambientes estressantes, baixa atividade física e sedentarismo ^2^. No Brasil, estudo transversal^5^ sobre consumo alimentar da Pesquisa de Orçamentos Familiares 2008-2009 em amostra representativa da população de 10 ou mais anos de idade observou que o maior consumo de alimentos ultraprocessados foi associado a maior teor de gorduras em geral, de gordura saturada, de gordura *trans* e de açúcar livre. Além disso, foi observado menor consumo de fibras, proteínas, sódio e potássio, quando comparado à fração do consumo relativa a alimentos *in natura* ou minimamente processados^5^.

Este suplemento da *Revista de Saúde Pública* apresenta dados do Estudo dos Riscos Cardiovasculares em Adolescentes (ERICA), que permitem avaliar as prevalências de hipertensão e vários fatores de risco cardiovascular em adolescentes no cenário brasileiro.

Os dados são de inegável importância para o planejamento e avaliação de políticas públicas necessárias e também para o desenvolvimento de ações mais específicas relacionadas ao ambiente escolar e à atenção primária, visando a redução dos vários agravos descritos para os adolescentes, que vão do consumo alimentar inadequado ao tabagismo e experimentação de álcool.

A importância de intervenções nessa fase do ciclo de vida decorre das grandes mudanças fisiológicas pelas quais os adolescentes passam, como ocorre na composição corporal, com ganho de gordura corporal mais expressivo entre meninas e com ganho de massa muscular duas vezes maior entre meninos^7^. Essas características demandam adequação da alimentação e suporte psicológico para o crescimento e desenvolvimento apropriados. Paralelamente a essas transformações fisiológicas, os adolescentes têm grande chance de adquirir hábitos alimentares inadequados e, em diferentes países, observam-se práticas alimentares não saudáveis neste grupo etário. Assim, adolescentes apresentam elevado consumo de alimentos industrializados, incluindo salgadinhos, biscoitos, bebidas açucaradas e demais alimentos com alta densidade calórica, além de tendência de baixo consumo de frutas e hortaliças, marcadores de alimentação saudável^4^ ^,^ ^8^.

Adicionalmente, os adolescentes vêm alterando progressivamente o consumo de refeições, com frequente omissão do desjejum e substituição de almoço e jantar por lanches não saudáveis, mudanças comportamentais consideradas desfavoráveis à saúde^1^ ^,^ ^6^.

Os artigos deste Suplemento avaliam as prevalências de hipertensão e excesso de peso em adolescentes, bem como outros fatores de risco cardiovascular, com destaque para características do consumo alimentar e atividade física. Os resultados foram estratificados segundo regiões do País, tipo de escola e características das cidades.

Este Suplemento apresenta leitura imprescindível para os estudiosos de doenças crônicas e da adolescência. Também permite vislumbrar, pela quantidade e qualidade dos dados obtidos, material sobre a saúde dos adolescentes para estudos futuros mais detalhados, que viabilizarão as necessárias e urgentes medidas de prevenção de doenças crônicas não transmissíveis e agravos que se iniciam na adolescência e persistem na vida adulta. Adicionalmente, o ERICA agregou grande número de pesquisadores que provavelmente trabalharão por condições de vida mais adequadas para os jovens.
